# Economic Impact of Progression from Mild Cognitive Impairment to Alzheimer Disease in the United States

**DOI:** 10.14283/jpad.2024.68

**Published:** 2024-04-02

**Authors:** Feride H. Frech, G. Li, T. Juday, Y. Ding, S. Mattke, A. Khachaturian, A.S. Rosenberg, C. Ndiba-Markey, A. Rava, R. Batrla, S. De Santi, H. Hampel

**Affiliations:** 1 U.S. HEOR & RWE (Health Economics, Outcomes Research & Real World Evidence) Eisai Inc., 200 Metro Blvd., 07110, Nutley, NJ, USA; 2 Genesis Research Group, Hoboken, NJ, USA; 3 The USC Brain Health Observatory, University of Southern California, Los Angeles, CA, USA; 4 Brain Watch Coalition/The Campaign to Prevent Alzheimer's Disease, Rockville, MD, USA; 5 Genworth, Richmond, VA, USA

**Keywords:** Mild cognitive impairment, Alzheimer disease, cost, burden of illness

## Abstract

**Background:**

Limited evidence exists on the economic burden of individuals who progress from mild cognitive impairment (MCI) to Alzheimer disease and related dementia disorders (ADRD).

**Objectives:**

To assess the all-cause health care resource utilization and costs for individuals who develop ADRD following an MCI diagnosis compared to those with stable MCI.

**Design:**

This was a retrospective cohort study from January 01, 2014, to December 31, 2019.

**Setting:**

The Merative MarketScan Commercial and Medicare Databases were used.

**Participants:**

Individuals were included if they: (1) were aged 50 years or older; (2) had ≥1 claim with an MCI diagnosis based on the International Classification of Diseases, Ninth Revision (ICD-9) code of 331.83 or the Tenth Revision (ICD-10) code of G31.84; and had continuous enrollment. Individuals were excluded if they had a diagnosis of Parkinson's disease or ADRD or prescription of ADRD medication.

**Measurements:**

Outcomes included all-cause utilization and costs per patient per year in the first 12 months following MCI diagnosis, in total and by care setting: inpatient admissions, emergency department (ED) visits, outpatient visits, and pharmacy claims.

**Results:**

Out of the total of 5185 included individuals, 1962 (37.8%) progressed to ADRD (MCI-to-ADRD subgroup) and 3223 (62.2%) did not (Stable MCI subgroup). Adjusted all-cause utilization was higher for all care settings in the MCI-to-ADRD subgroup compared with the Stable MCI subgroup. Adjusted all-cause mean total costs ($34599 vs $24541; mean ratio [MR], 1.41 [95% CI, 1.31–1.51]; P<.001), inpatient costs ($47463 vs $38004; MR, 1.25 [95% CI, 1.08–1.44]; P=.002), ED costs ($4875 vs $3863; MR, 1.26 [95% CI, 1.11–1.43]; P<.001), and outpatient costs ($16652 vs $13015; MR, 1.28 [95% CI, 1.20–1.37]; P<.001) were all significantly higher for the MCI-to-ADRD subgroup compared with the Stable MCI subgroup.

**Conclusions:**

Individuals who progressed from MCI to ADRD had significantly higher health care costs than individuals with stable MCI. Early identification of MCI and delaying its progression is important to improve patient and economic outcomes.

## Introduction

Alzheimer disease (AD) symptoms and severity are variable and range from a prodromal stage to late-stage dementia. Among individuals with dementia, mild cognitive impairment (MCI) is the first symptomatic stage on this non-linear continuum (1, 2). Even so, anosognosia rates as high as 60% have been reported (3). Fewer than 1 in 5 Americans are familiar with MCI (2), while up to 18% of the US population aged ≥60 years are living with MCI. AD is the most common cause of sporadic, age-related dementia disorders, but AD and related dementia disorders (ADRD) encompass other conditions such as Dementia with Lewy Bodies (4, 5). Not all individuals with MCI go on to develop AD or other forms of dementia disorders (6, 7). The cause of MCI is highly variable and can be due to AD, other types of neurologic disorders or primary neurodegenerative diseases, or secondary causes, such as certain medications and medical conditions (7, 8, 9). In some cases, MCI is reversible, such as MCI due to sleep and mood disorders. The 3-year progression rate of MCI to AD has been reported to be as high as 61% (10), with variability depending on diagnostic criteria, data source, and clinical setting.

An estimated 6.5 million (10.7%) Americans aged ≥65 years have AD dementia and 5.7 million (9.8%) have MCI due to AD (1, 11). Direct annual medical costs for individuals with ADRD aged ≥65 years in the US have been estimated at $321 billion, with 45% covered by Medicare and 19% covered by Medicaid (1). A substantial proportion of costs (25%) are paid by individuals out-of-pocket (1). Medicare is available for Americans aged ≥65, people with disabilities, and people with end-stage renal disease (12). Medicaid eligibility is based on income level and family size and can be modified by individual states (13).

Limited evidence exists examining the incremental economic burden of individuals who progress from MCI to ADRD. Identification and appropriate diagnosis of individuals at the early clinical and disease stages, such as MCI, and delaying progression could result in improved patient health and economic outcomes. This study aimed to assess the all-cause health care resource utilization, health care costs, and time to progression for individuals who develop ADRD following an MCI diagnosis.

## Methods

### Data Sources

This retrospective cohort study used the MarketScan Commercial and Medicare Databases and had an observation period of January 01, 2014, through December 31, 2019. These databases represent the health services of employees, dependents, and retirees in the US with primary or Medicare coverage through privately insured fee-for-service, point-of-service, or capitated health plans. All enrollment records and inpatient, outpatient, ancillary, and drug claims were collected. MarketScan is a registered trademark of Merative Corporation in the US, other countries, or both.

As the study did not constitute human subjects research per US federal regulations (45 CFR 46, 102(f))20, it was exempt from IRB review, consent requirements, and registration. Used primarily for research, the MarketScan Commercial and Medicare Databases are fully compliant with US privacy laws and regulations (i.e., HIPAA).

### Study Population and Participants

The index date was defined as the earliest date with a claim for MCI. Individuals were included if they: (1) were aged ≥50 years in the year of the index date; (2) had ≥1 claim with an MCI diagnosis based on the International Classification of Diseases, Ninth Revision (ICD-9) code of 331.83 or Tenth Revision (ICD-10) code of G31.84 between January 01, 2016, through December 31, 2018 (patient identification period); and (3) had continuous health plan enrollment ≥2 years before the index date and ≥1 year after the index date. A baseline period of 2 years prior to the index date was used to obtain as complete a medical history as possible. As part of another study, individuals were matched with controls with no MCI or ADRD diagnosis (14, 15).

Individuals were excluded if they had ≥1 claim with a diagnosis of Parkinson's disease any time during the study period or ≥1 claim with a diagnosis of ADRD any time prior to the index date. Individuals were also excluded if they had ≥1 pharmacy claim for an ADRD standard-of-care medication any time during the 2 years prior to the index date.

This study analyzed progression from MCI to ADRD instead of AD dementia alone to avoid underrepresentation due to inaccurate diagnoses (16). ADRD was defined by ICD-9 or ICD-10 codes related to ADRD (AD, Lewy-body associated dementia, frontotemporal dementia, vascular dementia and nonspecific dementias [eTables 1, 2, 3, and 4]) and/or ADRD medication use (donepezil, memantine, memantine/donepezil, galantamine, or rivastigmine). Among those who progressed to ADRD (MCI-to-ADRD subgroup), time to ADRD was calculated as the time from the index date to the first date of ADRD diagnosis and/or use of an ADRD medication, whichever came first. Stable MCI was defined as no ADRD diagnosis or medication use ≥2 weeks after initial MCI diagnosis.

### Variables and Outcomes

Demographic and clinical characteristics included age at index, categorical age (50 to 64, 65 to 79, and ≥80 years), sex, geographic region, comorbidity burden (using the Charlson Comorbidity Index [CCI] and Elixhauser Comorbidity Index [ECI], with a higher CCI or ECI representing a greater burden), comorbidities of interest, and brain imaging (computed tomography [CT] or magnetic resonance imaging [MRI]) data in the 90 days prior to or after index. Time to first ADRD diagnosis or medication (in months) was also assessed.

Outcomes included all-cause utilization and costs per patient per year (PPPY) in the first 12 months post-index date in total and by care setting: inpatient admissions, emergency department (ED) visits, outpatient visits, and pharmacy claims. Results for utilization are reported for all individuals and for individuals with ≥1 encounter, for each care setting. Cost results are reported only for individuals with ≥1 encounter for each care setting.

### Statistical Analysis

Demographics, clinical characteristics, brain imaging, and time to ADRD were summarized using descriptive statistics. Categorical measures were compared for the MCI-to-ADRD and Stable MCI subgroups using chi-square tests and continuous measures were compared using t-tests. Prevalence odds ratios with 95% CI were calculated for comorbidities. All-cause utilization PPPY in the 12 months post-index were estimated using generalized linear models (GLMs) including subgroups, age group, and sex with a Poisson distribution and log link. GLMs with a Gamma distribution and log link function were used for costs (17). The means and mean ratios with 95% CI were reported. All cost results were adjusted to December 2019 levels using the Medical Care component of the Consumer Price Index (CPI) (18).

GLMs offer a class of regression models that have a distribution in the exponential family. We used GLMs with Poisson distribution and loglink for our analyses of utilization, as they are often used for count data (e.g., outpatient visits) where the data are frequently skewed and have a zero cluster. We used GLMs with Gamma distribution and loglink for cost data as these data are non-negative and tend to be skewed to the right, with a large portion of observations having low expenditures but some having very large expenditures (17).

All analyses were conducted using SAS version 9.4.

### Sensitivity Analysis

A sensitivity analysis was conducted to understand the impact of variations in how ADRD was identified. This analysis included the following definitions from the MCI-to-ADRD subgroup: (a) MCI-to-ADRD diagnosis code with medication (received an ADRD diagnosis and used an ADRD medication ≥2 weeks after initial MCI diagnosis); (b) MCI-to-ADRD diagnosis code without medication (received an ADRD diagnosis and did not use an ADRD medication ≥2 weeks after initial MCI diagnosis); and (c) MCI-to-ADRD medication without diagnosis code (used an ADRD medication and did not receive an ADRD diagnosis ≥2 weeks after initial MCI diagnosis). These definitions were compared using the Stable MCI subgroup as the reference group.

## Results

### Demographics and Clinical Characteristics

**Figure 1:**
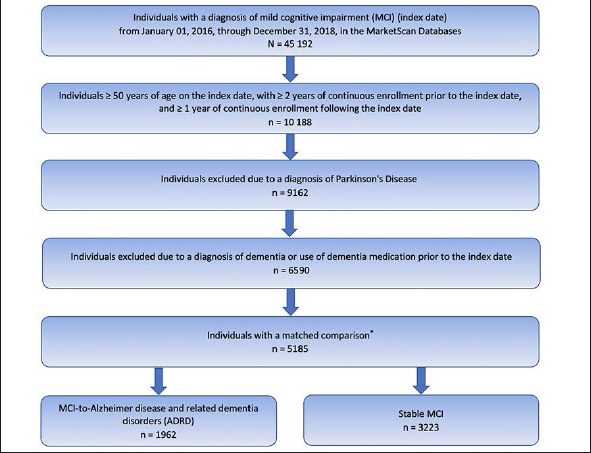
Study Population Identification * Mild cognitive impairment cohort for this study was determined by the number of individuals available for a matched control cohort for another study (14, 15).

A total of 5185 individuals met the inclusion and exclusion criteria (Figure 1). Of these, 1962 (37.8%) individuals progressed to ADRD (MCI-to-ADRD subgroup) and 3223 (62.2%) did not (Stable MCI subgroup). The mean (SD) time to ADRD was 9.55 (9.98) months.

The mean age for individuals in the overall MCI cohort was 67.0 years and 57.7% were female. The MCI-to-ADRD subgroup had a higher mean age than the Stable MCI subgroup (72.6 years vs 63.5 years; P<.001) (Table 1). The proportion of individuals in the 3 age groups (50 to 64, 65 to 79, and ≥80) in the MCI-to-ADRD subgroup was similar (Table 1). In contrast, a higher proportion of individuals (66.8%) in the Stable MCI subgroup were in the 50 to 64-year group. Within the MCI-to-ADRD subgroup, 56.0% were female compared to 58.7% within the Stable MCI subgroup (P=.06) (Table 1).

**Table 1:** Baseline Demographics and Clinical Characteristics*

Baseline characteristic	MCI-to-ADRD	Stable MCI	P value
Total, No. (%)	1962 (100.0)	3223 (100.0)	
Age at index (continuous), years			<.001
Mean (SD)	72.6 (11.9)	63.5 (10.7)	
Age (categorical), years, No. (%)			<.001
50–64	627 (32.0)	2154 (66.8)	
65–79	660 (33.6)	715 (22.2)	
≥ 80	675 (34.4)	354 (11.0)	
Sex, No. (%)			.06
Female	1099 (56.0)	1892 (58.7)	
Male	863 (44.0)	1331 (41.3)	
Region, No. (%)			.05
Northeast	627 (32.0)	944 (29.3)	
North Central	337 (17.2)	540 (16.8)	
South	691 (35.2)	1233 (38.3)	
West	302 (15.4)	504 (15.6)	
Other/Unknown	5 (0.3)	2 (0.1)	
Charlson Comorbidity Index			<.001
Mean (SD)	1.75 (1.66)	1.40 (1.50)	
Elixhauser Comorbidity Index			<.001
Mean (SD)	2.97 (2.18)	2.43 (2.03)	
Baseline comorbidities of interest, No. (%)			Odds ratio (95% CI)
Individuals with ≥1 comorbidity	1899 (96.8)	3057 (94.8)	1.64 (1.22–2.20)
Hypertension	1429 (72.8)	1950 (60.5)	1.75 (1.55–1.98)
Hyperlipidemia	1404 (71.6)	2019 (62.6)	1.50 (1.33–1.69)
Depression	597 (30.4)	1025 (31.8)	0.94 (0.83–1.06)
Ischemic heart disease	536 (27.3)	601 (18.6)	1.64 (1.44–1.87)
Chronic pulmonary disease	522 (26.6)	735 (22.8)	1.23 (1.08–1.40)
Diabetes	508 (25.9)	801 (24.9)	1.06 (0.93–1.20)
Stroke/transient ischemic attack	497 (25.3)	594 (18.4)	1.50 (1.31–1.72)
Obstructive sleep apnea	480 (24.5)	960 (29.8)	0.76 (0.67–0.87)
Hearing loss	470 (24.0)	620 (19.2)	1.32 (1.15–1.51)
Hypothyroidism	469 (23.9)	829 (25.7)	0.91 (0.80–1.03)
Obesity	298 (15.2)	630 (19.5)	0.74 (0.63–0.86)
Atherosclerosis	262 (13.4)	257 (8.0)	1.78 (1.48–2.13)
Insomnia	258 (13.1)	500 (15.5)	0.82 (0.70–0.97)
Atrial fibrillation	253 (12.9)	245 (7.6)	1.80 (1.49–2.17)
Congestive heart failure	204 (10.4)	211 (6.5)	1.66 (1.35–2.03)
Weight loss	153 (7.8)	167 (5.2)	1.55 (1.23–1.94)
Myocardial infarction	120 (6.1)	111 (3.4)	1.83 (1.40–2.38)
Metabolic syndrome	114 (5.8)	162 (5.0)	1.17 (0.91–1.49)
Chronic kidney disease	101 (5.1)	103 (3.2)	1.64 (1.24–2.18)
Alcohol abuse	48 (2.4)	88 (2.7)	0.89 (0.63–1.28)
Drug abuse	48 (2.4)	94 (2.9)	0.83 (0.59–1.19)
Bipolar disorder	44 (2.2)	76 (2.4)	0.95 (0.65–1.38)
Psychosis	29 (1.5)	44 (1.4)	1.08 (0.68–1.74)
Disturbances of sensation of smell and taste	12 (0.6)	23 (0.7)	0.86 (0.43–1.72)
Schizophrenia	7 (0.4)	14 (0.4)	0.82 (0.33–2.04)
Brain imaging, No. (%)			
Individuals with ≥1 CT	1028 (52.4)	579 (18.0)	NA
Individuals with ≥1 MRI	1220 (62.2)	1101 (34.2)	NA
Individuals with ≥1 CT or MRI	1525 (77.7)	1265 (39.2)	NA


Abbreviations: ADRD, Alzheimer disease and related dementia disorders; CT, computed tomography; MCI, mild cognitive impairment; MRI, magnetic resonance imaging; NA, not applicable; SD, standard deviation. * Because of rounding, percentages may not total 100.

The MCI-to-ADRD subgroup had a significantly higher comorbidity burden compared with the Stable MCI subgroup, with a higher mean CCI (1.75 vs 1.40; P<.001) and ECI (2.97 vs 2.43; P<.001) (Table 1). Of the comorbidities of interest, hypertension, hyperlipidemia, and depression were the most frequently reported in both subgroups. Compared with the Stable MCI subgroup, the MCI-to-ADRD subgroup had significantly higher proportions of hypertension (72.8% vs 60.5%; odds ratio [OR], 1.75 [95% CI, 1.55–1.98]) and hyperlipidemia (71.6% vs 62.6%; OR, 1.50 [95% CI, 1.33–1.69]). The Stable MCI and the MCI-to-ADRD subgroups had similar proportions of individuals with a diagnosis of depression (30.4% vs 31.8%; OR, 0.94 [95% CI, 0.83–1.06]) (Table 1). In the MCI-to-ADRD subgroup, 77.7% (1525) of individuals had ≥1 a brain CT or MRI compared with 39.2% (1265) in the Stable MCI subgroup (Table 1).

### All-Cause Health Care Resource Utilization

Adjusted all-cause utilization was higher for all care settings in the MCI-to-ADRD subgroup compared with the Stable MCI subgroup (Table 2). All-cause inpatient utilization was greater in the MCI-to-ADRD subgroup compared with the Stable MCI subgroup (0.33 vs 0.18; mean ratio [MR], 1.88; 95% CI, 1.66-2.13; P<.001). A higher proportion of the MCI-to-ADRD subgroup had ≥1 inpatient admission compared with the Stable MCI subgroup (22.7% vs 12.8%). The mean length of stay in days was longer for the MCI-to-ADRD subgroup (1.19 vs 0.67; P<.001). All-cause ED visits were greater in the MCI-to-ADRD subgroup compared with the Stable MCI subgroup (0.79 vs 0.43; MR, 1.83; 95% CI, 1.69–1.98; P<.001). A higher proportion of the MCI-to-ADRD subgroup had ≥1 ED visit compared with the Stable MCI subgroup (38.5% vs 23.9%). The mean number of outpatient visits was significantly higher in the MCI-to-ADRD subgroup (30.84 vs 25.65; P<.001). While the proportion of individuals with ≥1 pharmacy claim was similar in the MCI-to-ADRD and Stable MCI subgroups (96.5% vs 93.9%), the mean number of pharmacy claims was significantly higher in the MCI-to-ADRD subgroup (27.19 vs 23.43; P<.001) (Table 2). Of the individuals who progressed to ADRD, 54.1% of individuals had a pharmacy claim for an ADRD medication.

**Table 2:** Adjusted All-Cause Health Care Resource Utilization in the 12-months Post-Index by Care Setting, Adjusted for Age Group and Sex*

Category	MCI-to-ADRD	Stable MCI	P value
Total, No. (%)	1962 (100.0)	3223 (100.0)	
Inpatient admissions			
Admissions			
Mean	0.33	0.18	<.001
Mean ratio (95% CI)	1.88 (1.66–2.13)	Ref.	
Length of stay (days)			
Mean	1.19	0.67	<.001
Mean ratio (95% CI)	1.78 (1.67–1.89)	Ref.	
**Individuals with ≥1 inpatient admission, No. (%)**	446 (22.7)	413 (12.8)	
Admissions			
Mean	1.47	1.23	.004
Mean ratio (95% CI)	1.20 (1.06–1.35)	Ref.	
Length of stay (days)			
Mean	5.30	4.69	<.001
Mean ratio (95% CI)	1.13 (1.06–1.21)	Ref.	
ED visits			
Mean	0.79	0.43	<.001
Mean ratio (95% CI)	1.83 (1.69–1.98)	Ref.	
**Individuals with ≥1 ED visit, No. (%)**	756 (38.5)	771 (23.9)	
Mean	2.07	1.75	<.001
Mean ratio (95% CI)	1.18 (1.09–1.28)	Ref.	
Outpatient visits			
Mean	30.84	25.65	<.001
Mean ratio (95% CI)	1.20 (1.19–1.22)	Ref.	
**Individuals with ≥1 outpatient visit, No. (%)**	1962 (100.0)	3223 (100.0)	
Mean	30.84	25.65	<.001
Mean ratio (95% CI)	1.20 (1.19–1.22)	Ref.	
Pharmacy			
Mean	26.21	21.93	<.001
Mean ratio (95% CI)	1.20 (1.18–1.21)	Ref.	
**Individuals with ≥1 prescription claim, No. (%)**	1893 (96.5)	3028 (93.9)	
Mean	27.19	23.43	<.001
Mean ratio (95% CI)	1.16 (1.15–1.17)	Ref.	


Abbreviations: ADRD, Alzheimer disease and related dementia disorders; CI, confidence interval; ED, emergency department; MCI, mild cognitive impairment; Ref., reference; * Means are estimated using a generalized linear model.

### All-Cause Health Care Costs

**Figure 2:**
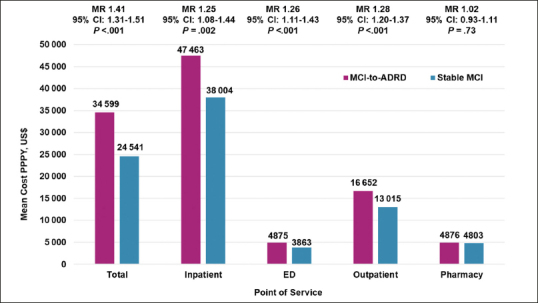
Adjusted All-Cause Mean Costs Per Patient Per Year in the 12-Months Post-Index for Individuals with ≥1 Encounter by Care Setting and Adjusted for Age Group and Sex*,† Abbreviations: ADRD, Alzheimer disease and related dementia disorders; MCI, mild cognitive impairment; MR, mean ratio; PPPY, per patient per year; US, United States; * Mean costs are calculated for individuals with ≥1 encounter for each care setting. All results were inflated to December 2019 United States Dollars based on the Medical Care cost component of the Consumer Price Index. † Means are estimated using a generalized linear model.

Adjusted all-cause mean total costs were significantly higher ($34599 vs $24541; MR, 1.41 [95% CI, 1.31–1.51]; P<.001) for the MCI-to-ADRD subgroup compared with the Stable MCI subgroup. Inpatient costs ($47 463 vs $38004; MR, 1.25 [95% CI, 1.08–1.44]; P=.002), ED costs ($4875 vs $3863; MR, 1.26 [95% CI, 1.11–1.43]; P<.001), and outpatient costs ($16652 vs $13015; MR, 1.28 [95% CI, 1.20–1.37]; P<.001) were all significantly higher for the MCI-to-ADRD subgroup compared with the Stable MCI subgroup. Pharmacy costs were higher among the MCI-to-ADRD subgroup compared with the Stable MCI subgroup, but the difference was not statistically significant ($4876 vs $4803; MR, 1.02 [95% CI, 0.93-1.11]; P=.73). Adjusted all-cause mean costs PPPY for individuals with ≥1 encounter were significantly higher for all care settings in the MCI-to-ADRD subgroup compared with the Stable MCI subgroup, except for pharmacy costs (Figure 2; Table 3).

**Table 3:** Adjusted All-Cause Mean Costs Per Patient Per Year in the 12-Months Post-Index for Individuals with ≥1 Encounter by Care Setting and Adjusted for Age Group and Sex*,†

Category	MCI-to-ADRD	Stable MCI	P value
Total, No. (%)	1962 (100.0)	3223 (100.0)	
Total cost			
Individuals with ≥1 encounter, No. (%)	1962 (100.0)	3223 (100.0)	
Mean ($)	$34599	$24541	<.001
Mean ratio (95% CI)	1.41 (1.31–1.51)	Ref.	
Inpatient cost			
Individuals with ≥1 inpatient admission, No. (%)	446 (22.7)	413 (12.8)	
Mean ($)	$47463	$38004	.002
Mean ratio (95% CI)	1.25 (1.08–1.44)	Ref.	
Emergency department cost			
Individuals with ≥1 ED visit, No. (%)	756 (38.5)	771 (23.9)	
Mean ($)	$4875	$3863	<.001
Mean ratio (95% CI)	1.26 (1.11–1.43)	Ref.	
Outpatient cost			
Individuals with ≥1 outpatient visit, No. (%)	1962 (100.0)	3223 (100.0)	
Mean ($)	$16652	$13015	<.001
Mean ratio (95% CI)	1.28 (1.20–1.37)	Ref.	
Pharmacy cost			
Individuals with ≥1 prescription claim, No. (%)	1893 (96.5)	3028 (93.9)	
Mean ($)	$4876	$4803	.73
Mean ratio (95% CI)	1.02 (0.93–1.11)	Ref.	


Abbreviations: ADRD, Alzheimer disease and related dementia disorders; ED, emergency department; MCI, mild cognitive impairment; Ref., reference; * All results were inflated to December 2019 United States Dollars based on the Medical Care cost component of the Consumer Price Index and rounded to the nearest dollar. † Means are estimated using a generalized linear model.

### Sensitivity Analysis

For individuals in the MCI-to-ADRD subgroup, demographics and patient characteristics were similar for all 3 different definitions used for detecting ADRD (eTable 5). Costs were higher for each of the patient groups identified with the different definitions of MCI-to-ADRD compared with the Stable MCI subgroup – diagnosis only ($41 478 vs $24597; P<.001), medication only ($31342 vs $25558; P=.007), and diagnosis and medication ($27331 vs $25379; P=.15) (eTable 6).

## Discussion

In this health insurance claims study, the group that progressed incurred more costs with 41% higher overall costs than non-progression, an incremental cost of over $10000, in the 12 months following a diagnosis of MCI. Individuals with MCI, who progressed to ADRD were older at diagnosis than individuals with stable MCI, had a higher comorbidity burden, consumed more health care resources, and incurred higher health care costs. These cost differences were primarily driven by higher inpatient costs. No statistically significant difference in pharmacy costs was observed between individuals who progressed from MCI to ADRD and those with stable MCI. Just over half of the individuals who developed ADRD during the follow-up period had a pharmacy claim for an ADRD medication.

More than one-third of individuals in this study progressed to ADRD within a year of their first MCI diagnosis. Reported rates of progression from diagnosed MCI to ADRD in the published literature are variable depending on criteria and the database used for diagnosis, duration of the study, and the clinical setting; the rate of progression in our study of 38% is consistent with the 36% that was found in another study using Medicare health insurance claims data (19).

Mean time to ADRD diagnosis in our study was close to 10 months. This finding may indicate that some MCI individuals actually had misdiagnosed mild dementia. Even so, the findings are generally consistent with other published research (20, 21, 22) but variability exists in reported time to ADRD depending on how the diagnosis is made, progression is defined, and the sociodemographic characteristics of the study population. One Swedish study (n=21), where individuals were recruited after referral for investigation of suspected dementia, reported one-third of individuals converted from MCI to AD in 8.1 months (20). In a US study, mean time from MCI diagnosis to progression to a dementia syndrome was reported to be 2.19 years, but rates varied widely depending on source of patient referral (21).

While the economic burden of ADRD is well established (1, 19, 23), less is known about the economic burden of the progression from MCI to ADRD. Our findings are consistent with a prior study that found individuals with MCI who later progressed to ADRD had higher Medicare expenditures than individuals with MCI who did not progress in the 12 months after MCI diagnosis, with inpatient care being the main expenditure throughout the disease process (19). Inpatient costs were the main driver of overall costs in our study, and this is similar to what has been reported in other studies (19, 24, 25). The results of our study are also consistent with previously published estimates of utilization, as published literature has shown increased hospitalization rates and ED utilization in individuals with dementia compared with individuals without dementia (26, 27).

More than one-half of individuals with MCI who progressed to ADRD were treated with a medication in our study, similar to what has been reported by the National Institute for Health and Care Excellence (NICE) Technology Appraisals in the United Kingdom (28). With the introduction of biologics, treatment patterns may change and as this landscape evolves, the timely diagnosis of MCI and ADRD will become even more important.

Delays in diagnosis of MCI and AD can limit access to interventions, coordinated programs, and pharmacologic treatment and affect cost of care (29, 30). One study found that the benefits of early identification and treatment of individuals with AD were highest when diagnosis was made at the earlier stages and when pharmacologic therapy was combined with caregiver support (31). Lifestyle interventions such as exercise and diet can also be important in delaying progression (32). A global panel was convened in April 2019 to come to an expert consensus on the screening, identification, and management of MCI (33, 34, 35). Consistent diagnostic criteria for MCI have also been identified as an important step towards improving care for individuals with MCI (36).

It is also important to note that direct medical costs for individuals with MCI and ADRD are substantial, higher than similar individuals without these conditions (19) and likely to increase. The total costs associated with MCI and ADRD are expected to increase as the US population ages and the number of people in the US with MCI is projected to increase 76.2% by 2060 (37) and 178% for those with ADRD (38). The results of our study support the importance of improved recognition and detection of MCI and AD symptoms in order to improve both patient and economic outcomes.

### Limitations

The results of this study should be considered within the context of some limitations. Data from the Merative MarketScan Commercial and Medicare Databases come from employers, health plans, hospitals, and Medicare and Medicaid programs; findings may not be generalizable to the uninsured or underinsured populations. Although the MarketScan Databases cover geographically diverse US regions with a broad range of ages, the results may not be generalizable to the entire US population. Health insurance claims data are collected for reimbursement and not research purposes and, as such, subject to missing important clinical covariates such as severity of illness and to coding errors.

The results of this study were adjusted for age group and sex but not for other demographic or clinical factors, such as the baseline comorbidity index. This variable, in particular, may impact the differences observed in diagnosis, utilization, and costs as the MCI-to-ADRD subgroup had a higher comorbidity burden at baseline than the Stable MCI subgroup. While longitudinal, our observational study design precludes assessment of causality. We excluded patients with a diagnosis of Parkinson's disease because, while dementia is a common comorbidity in patients with the disease (39, 40, 41), the onset of dementia in these patients usually occurs years after the original diagnosis and our area of interest was on patients who were diagnosed with MCI and had no additional known risk factors (39, 41, 42).

Multiple factors can contribute to the underdiagnosis of ADRD and MCI including a practitioner's level of disease awareness, knowledge, comfort, and certainty in making the diagnosis; the complexity of and access to testing; lack of recognition of the symptoms by individuals and care partners; and socioeconomic differences. A survey of 801 US primary care physicians (PCPs) conducted in 2021 for the Alzheimer's Association found that 51% of PCPs were not fully comfortable diagnosing MCI due to AD (1). One study also found that PCPs only diagnose MCI at a rate of 6% of individuals (22, 43). Another study of the Medicare population found the rate of MCI diagnosis to be 7.9% (22). Furthermore, a 2018 study of adults aged 65 years and older with probable dementia found that 39.5% were undiagnosed and 19.2% were unaware of the diagnosis (44). Given the underreporting and underdiagnosis of MCI, this analysis may not represent all individuals with MCI and may underestimate the true burden of MCI. Future research should assess the robustness of the results, given the current limitations discussed.

## Conclusions

Compared to individuals with stable MCI, individuals with MCI who progress to dementia incurred more costs primarily because of inpatient visits. Individuals with MCI who progressed to dementia had higher comorbidity burden. Beyond increased health care costs from Medicare, the economic and societal impact of unreimbursed dementia care is enormous and often crushing for patients and family members. As with most medical conditions, early identification and appropriate diagnosis are important to improve patient and economic outcomes. New treatments that better manage MCI may change the course of the disease. Future research should examine the impact of these new treatments on utilization and costs.

## Electronic Supplementary Material

Supplementary material, approximately 49.9 KB.

*Funding:* This study was funded and sponsored by Eisai Inc. The sponsor was involved in the design of the study, the analysis and interpretation of the results, and the preparation and final approval of the manuscript.

*Conflict of Interest Statements:* Feride H Frech is an employee of Eisai Inc. Gang Li is an employee of Eisai Inc. Timothy Juday is an employee of Eisai Inc. Yingjie Ding is an employee of Genesis Research Group. Soeren Mattke serves on the board of directors of Senscio Systems, Inc., and the scientific advisory board of AiCure Technologies, Alzpath, and Boston Millennia Partners and has received consulting and speaker fees from Biogen, C2N, Eisai, Novartis, Novo Nordisk and Roche/Genentech. Ara Khachaturian is an Officer and director of the Campaign to Prevent Alzheimer's Disease (PAD 20/20) and; Officer, director and employee of Khachaturian and Associates; Founding executive-editor of Alzheimer's & Dementia, The Journal of the Alzheimer's Association (retired), Founding executive-editor of Alzheimer's & Dementia: Translational Research & Clinical Intervention (retired), Founding executive-editor of Alzheimer's & Dementia: Diagnoses, Assessment & Disease Monitoring (retired); Executive Officer and Director, Brain Watch Coalition; Senior Research Fellow, University of Nevada Las Vegas, National Supercomputing Institute & Dedicated Research Network; Received payments through organizational affiliations for grants, contracts, consulting fees, honoraria, meeting support, travel support, in-kind research/professional support over the last 36 months from the Alzheimer's Association, Acadia Pharmaceuticals, Alzheon, Biogen, Clinical Trials Alzheimer's Disease Conference, Davos Alzheimer's Consortium, Eisai, Eli Lilly & Company, RELX Plc, High Lantern Group, International Neurodegenerative Disorders Research Center, and Serdi Publishing. Aaron S Rosenberg has no financial conflicts of interest to disclose. Colette Ndiba-Markey is an employee of Genesis Research Group. Andrew Rava is an employee of Genesis Research Group. Richard Batrla is an employee of Eisai Inc. Susan De Santi is an employee of Eisai Inc. Harald Hampel is an employee of Eisai Inc. He serves as Reviewing Editor for the Journal Alzheimer's and Dementia, as Editorial Board Member of the Journal of Prevention of Alzheimer's Disease (JPAD), as Scientific Committee Member of the annual Clinical Trials on Alzheimer's Disease (CTAD) conference, and as Scientific Program Reviewer of the Alzheimer's Association International Conference (AAIC). He is inventor of 11 patents and has received no royalties: (i) In Vitro Multiparameter Determination Method for The Diagnosis and Early Diagnosis of Neurodegenerative Disorders Patent Number: 8916388; (ii) In Vitro Procedure for Diagnosis and Early Diagnosis of Neurodegenerative Diseases Patent Number: 8298784; (iii) Neurodegenerative Markers for Psychiatric Conditions Publication Number: 20120196300; (iv) In Vitro Multiparameter Determination Method for The Diagnosis and Early Diagnosis of Neurodegenerative Disorders Publication Number: 20100062463; (v) In Vitro Method for The Diagnosis and Early Diagnosis of Neurodegenerative Disorders Publication Number: 20100035286; (vi) In Vitro Procedure for Diagnosis and Early Diagnosis of Neurodegenerative Diseases Publication Number: 20090263822; (vii) In Vitro Method for The Diagnosis of Neurodegenerative Diseases Patent Number: 7547553; (viii) CSF Diagnostic in Vitro Method for Diagnosis of Dementias and Neuroinflammatory Diseases Publication Number: 20080206797; (ix) In Vitro Method for The Diagnosis of Neurodegenerative Diseases Publication Number: 20080199966; (x) Neurodegenerative Markers for Psychiatric Conditions Publication Number: 20080131921; and (xi) Method for diagnosis of dementias and neuroinflammatory diseases based on an increased level of procalcitonin in cerebrospinal fluid: Publication number: United States Patent 10921330.
