# Supplementary material for: Economic Impact of Progression from Mild Cognitive Impairment to Alzheimer Disease in the United States
Source: J Prev Alzheimers Dis. 2024 Apr 2;11(4):983–91. doi: 10.14283/jpad.2024.68 (PMC11266270; doi:10.14283/jpad.2024.68)
Supplement: Supplementary file 1 — Supplementary material, approximately 49.9 KB. [file mmc1.docx]

**Supplemental Material**

**eTable 1. List of International Classification of Diseases 9 and 10 Codes for Alzheimer Diseases**

**eTable 2. List of International Classification of Diseases 9 and 10 Codes for Non-Alzheimer Dementia**

**eTable 3. List of International Classification of Diseases 9 and 10 Codes for Unspecified Dementia**

**eTable 4. List of International Classification of Diseases 9 and 10 Codes for Vascular Dementia**

**eTable 5. Baseline Demographics and Clinical Characteristics of the Definitions of Alzheimer Disease and Related Dementia Disorders in the Sensitivity Analysis**

**eTable 6. Adjusted All-Cause Mean Costs Per Patient Per Year in the 12-Months Post-Index for Individuals with ≥1 Encounter of the Definitions of Alzheimer Disease and Related Dementia Disorders in the Sensitivity Analysis, by Care Setting and Adjusted for Age Group and Sex^a,b^**

**eTable 1. List of International Classification of Diseases 9 and 10 Codes for Alzheimer Diseases**

| **ICD-9 Description** | **ICD-9** | **ICD-10** | **ICD-10 Description** |
| --- | --- | --- | --- |
| Other cerebral degenerations | 331 |  |  |
| Alzheimer Disease | 331.0 | G30.0 | Alzheimer disease with early onset |
|  |  | G30.1 | Alzheimer disease with late onset |
|  |  | G30.8 | Other Alzheimer disease |
|  |  | G30.9 | Alzheimer disease, unspecified |

**Abbreviations:** ICD, International Classification of Diseases.

**eTable 2. List of International Classification of Diseases 9 and 10 Codes for Non-Alzheimer Dementia**

| **ICD-9 Description** | **ICD-9** | **ICD-10** | **ICD-10 Description** |
| --- | --- | --- | --- |
| Other and unspecified prion disease of central nervous system | 46.79 | A81.89 | Other atypical virus infections of central nervous system |
| Pre-senile dementia, uncomplicated | 290.1 |  |  |
| Presenile dementia | 290.10 |  |  |
| Pre-senile dementia with delusion features | 290.12 |  |  |
| Pre-senile dementia with depressive feature | 290.13 |  |  |
| Persistent mental disorders due to conditions classified elsewhere | 294 |  |  |
| Amnestic disord oth dis | 294.0 |  |  |
| Other persistent mental disorders due to conditions classified elsewhere | 294.8 |  |  |
| Mental disor NOS oth dis | 294.9 |  |  |
| Unspecified non-psychotic mental disorder following organic brain damage | 310.9 |  |  |
| Conduct disturbance NOS | 312.9 |  |  |
| Encephalopathy, not elsewhere classified | 348.3 |  |  |
| Nonpsych mntl disord NEC | 310.89 |  |  |
| Encephalopathy NOS | 348.30 |  |  |
| Encephalopathy NEC | 348.39 |  |  |
| Temporal sclerosis | 348.81 |  |  |
|  |  | F04 | Amnestic disorder due to known physiological condition Korsakov's psychosis or syndrome, nonalcoholic |
|  |  | F06.8 | Oth mental disorders due to known physiological condition |
|  |  | F07.89 | Other personality and behavioral disorders due to known physiological condition |
| Amyotrophic lateral sclerosis | 335.20 | G12.21 | Amyotrophic lateral sclerosis |
| Parkinson's disease | 331.82 | G20 | Parkinson's disease |
| Pick's disease | 331.11 | G31.01 | Pick's disease |
| Other frontotemporal dementia | 331.19 | G310.9 | Other frontotemporal dementia |
| Senile degeneration of | 331.12 |  |  |
| brain, not elsewhere |  |  |  |
| Classified |  |  |  |
| Dementia with Lewy | 331.82 | G31.83 | Dementia with Lewy |
| Bodies |  |  | Bodies |
|  |  | G31.84 | Mild cognitive impairment, so stated |
| Corticobasal degeneration | 331.6 | G31.85 | Corticobasal degeneration |
|  |  | G93.4 | Other and unspecified encephalopathy |
|  |  | G93.40 | Encephalopathy, unspecified |
|  |  | G93.49 | Other encephalopathy |
| Hippocampal sclerosis |  | G93.81 | Temporal sclerosis |
| Altered mental status | 780.97 |  |  |

**Abbreviations:** ICD, International Classification of Diseases.

**eTable 3. List of International Classification of Diseases 9 and 10 Codes for Unspecified Dementia**

| **ICD-9 Description** | **ICD-9** | **ICD-10** | **ICD-10 Description** |  |
| --- | --- | --- | --- | --- |
| Dementias | 290 | F03.90 | Unspecified dementia without behavioral disturbance |  |
| Senile dementia, uncomplicated | 290.0 |  |  |  |
| Pre-senile dementia, uncomplicated | 290.1 |  |  |  |
| Pre-senile dementia with | 290.12 |  |  |  |
| delusion features |  |  |  |  |
| Pre-senile dementia with depressive feature | 290.13 |  |  |  |
| Senile dementia with delusional features | 290.2 |  |  |  |
| Senile delusion | 290.20 |  |  |  |
| Senile dementia with depressive features | 290.21 |  |  |  |
| Senile delirium | 290.3 |  |  |  |
| Dementia, unspecified, without behavioral disturbance | 294.2 |  |  |  |
|  |  | F02.8 | Dementia in other diseases classified elsewhere |  |
| Dementia w/o behav dist | 294.10 |  |  |  |
| Dementia classified elsewhere without behavioral disturbance | 294.1 | F02.80 | Dementia in other diseases classified elsewhere without behavioral disturbance |  |
|  |  |  |  |  |
| Dementia classified elsewhere with behavioral disturbance | 294.11 | F02.81 | Dementia in other diseases classified elsewhere with behavioral disturbance |  |
|  |  |  |  |  |
|  |  | F03.9 | Unspecified dementia |  |
| Demen NOS w/o behv dstrb | 294.20 |  |  |  |
| Dementia, unspecified, with behavioral disturbance Aggressive behavior Combative behavior Violent behavior | 294.21 | F03.91 | Unspecified dementia with behavioral disturbance |  |
| Other persistent mental disorders due to conditions classified elsewhere | 294.8 | F06.0 | Psychotic disorder with hallucinations due to known physiological condition |  |
|  |  |  |  |  |
|  |  | G31.1 | Senile degeneration of brain, not elsewhere Classified |  |
| Senile degenerat brain | 331.2 |  |  |  |
| Other cerebral degenerations; Cerebral degeneration, unspecified | 331.9 | G31.9 | Degenerative disease of nervous system, unspecified |  |
|  |  |  |  |  |
| Unspecified non-psychotic mental disorder following organic brain damage | 310.9 | F09 | Unspecified mental disorder due to known physiological condition |  |
| Pseudobulbar affect | 310.81 | F48.2 | Pseudobulbar affect |  |
| Altered mental status | 780.97 | R41.82 | Altered mental status, unspecified |  |
| Delirium due to conditions classified elsewhere | 293.0 | F05 | Delirium due to known physiological condition |  |
| Wandering in diseases classified elsewhere | V40.31 | Z91.83 | Wandering in diseases classified elsewhere |  |

**Abbreviations:** ICD, International Classification of Diseases.

**eTable 4. List of International Classification of Diseases 9 and 10 Codes for Vascular Dementia**

| **ICD-9 Description** | **ICD-9** | **ICD-10** | **ICD-10 Description** |
| --- | --- | --- | --- |
| Vascular dementia uncomplicated | 290.4 | F01.50 | Vascular dementia without behavioral disturbance |
| Vascular dementia with delirium | 290.41 | F01.51 | Vascular dementia with behavioral disturbance |
| Delirium due to known physiological condition Vascular dementia with delusions | 290.42 |  |  |
| Vascular dementia with depression | 290.43 |  |  |
| Dementia following  hypoxic-ischemic injury | 348.1 | G93.1 | Anoxic brain damage, not elsewhere classified |
|  |  | I69.010 | Attention and concentration deficit following nontraumatic subarachnoid hemorrhage |
|  |  | I69.011 | Memory deficit following ntrm subarachnoid hemorrhage |
|  |  | I69.012 | Vis def/sptl nglct following ntrm subarachnoid hemorrhage |
|  |  | I69.013 | Visuospatial deficit and spatial neglect following nontraumatic subarachnoid hemorrhage |
|  |  | I69.014 | Frontal lobe and executive function deficit following nontraumatic subarachnoid hemorrhage |
|  |  | I69.015 | Cognitive social or emotional deficit following nontraumatic subarachnoid hemorrhage |
|  |  | I69.018 | Other symptoms and signs involving cognitive functions following nontraumatic subarachnoid hemorrhage |
|  |  | I69.019 | Unspecified symptoms and signs involving cognitive functions following nontraumatic subarachnoid hemorrhage |
| Late effects of  cerebrovascular disease,  cognitive deficits | 438.0 | I69.31 | Attention and concentration deficit following cerebral infarction |
|  |  | I69.311 | Memory deficit following cerebral infarction |
|  |  | I69.312 | Visuospatial deficit and spatial neglect following cerebral infarction |
|  |  | I69.313 | Psychomotor deficit following cerebral infarction |
|  |  | I69.314 | Frontal lobe and executive function deficit following cerebral infarction |
|  |  | I69.315 | Cognitive social or emotional deficit following cerebral infarction |
|  |  | I69.318 | Other symptoms and signs involving cognitive functions following cerebral infarction |
|  |  | I69.319 | Unspecified symptoms and signs involving cognitive functions following cerebral infarction |
| Unspecified late effects of cerebrovascular disease | 438.9 | I69.9 | Unspecified sequelae of unspecified cerebrovascular disease (Cognitive deficit due to preceding CVA) Late effects of cerebrovascular disease, cognitive deficits |

**Abbreviations:** ICD, International Classification of Diseases.

**eTable 5. Baseline Demographics and Clinical Characteristics of the Definitions of Alzheimer Disease and Related Dementia Disorders in the Sensitivity Analysis**

| **Baseline characteristic** | **Total**  **MCI-to-ADRD subgroup** | **MCI-to-ADRD Diagnosis code and medication** | **MCI-to-ADRD Diagnosis code without medication** | **MCI-to-ADRD Medication without diagnosis code** |
| --- | --- | --- | --- | --- |
| **Total** | **1962** | **775** | **900** | **287** |
| **Age at index (continuous)** |  |  |  |  |
| Mean (SD) | 72.6 (11.9) | 75.4 (10.2) | 70.7 (13.1) | 71.3 (11.2) |
| **Age (categorical), years, No. (%)** |  |  |  |  |
| 50-64 | 627 (32.0) | 146 (18.8) | 385 (42.8) | 96 (33.4) |
| 65-79 | 660 (33.6) | 316 (40.8) | 233 (25.9) | 111 (38.7) |
| ≥ 80 | 675 (34.4) | 313 (40.4) | 282 (31.3) | 80 (27.9) |
| **Sex, No. (%)** |  |  |  |  |
| Male | 863 (44.0) | 345 (44.5) | 389 (43.2) | 129 (44.9) |
| Female | 1099 (56.0) | 430 (55.5) | 511 (56.8) | 158 (55.1) |
| **Charlson Comorbidity Index** |  |  |  |  |
| Mean (SD) | 1.75 (1.66) | 1.62 (1.54) | 1.94 (1.79) | 1.47 (1.49) |
| **Elixhauser Comorbidity Index** |  |  |  |  |
| Mean (SD) | 2.97 (2.18) | 2.82 (2.06) | 3.24 (2.32) | 2.51 (1.89) |

**Abbreviations:** ADRD, Alzheimer disease and related dementia disorders; MCI, mild cognitive impairment; SD, standard deviation.

**eTable 6. Adjusted All-Cause Mean Costs Per Patient Per Year in the 12-Months Post-Index for Individuals with ≥1 Encounter of the Definitions of Alzheimer Disease and Related Dementia Disorders in the Sensitivity Analysis, by Care Setting and Adjusted for Age Group and Sex^*,†^**

| \| **Category** \| **MCI-to-ADRD Diagnosis code and medication** \| **Stable MCI** \| ***P* value** \| **MCI-to-ADRD Diagnosis code without medication** \| **Stable MCI** \| ***P* value** \| **MCI-to-ADRD**  **Medication without diagnosis code** \| **Stable MCI** \| ***P* value** \| \| --- \| --- \| --- \| --- \| --- \| --- \| --- \| --- \| --- \| --- \| \| **Total, No. (%)** \| **775 (100.0)** \| **3223 (100.0)** \|  \| **900 (100.0)** \| **3223 (100.0)** \|  \| **287 (100.0)** \| **3223 (100.0)** \|  \| \| **Total Cost** \|  \|  \|  \|  \|  \|  \|  \|  \|  \| \| **Individuals with ≥1 encounter, No. (%)** \| 775 (100.0) \| 3223 (100.0) \|  \| 900 (100.0) \| 3223 (100.0) \|  \| 287 (100.0) \| 3223 (100.0) \|  \| \| Mean ($) \| $27 331 \| $25 379 \| .15 \| $41 478 \| $24 597 \| <.001 \| $31 342 \| $25 558 \| .007 \| \| Mean ratio (95% CI) \| 1.08 (0.97-1.19) \| Ref. \|  \| 1.69 (1.54-1.84) \| Ref. \|  \| 1.23 (1.06-1.42) \| Ref. \|  \| \| **Inpatient cost** \|  \|  \|  \|  \|  \|  \|  \|  \|  \| \| **Individuals with ≥1 admission, No. (%)** \| 146 (18.8) \| 413 (12.8) \|  \| 263 (29.2) \| 413 (12.8) \|  \| 37 (12.9) \| 413 (12.8) \|  \| \| Mean ($) \| $42 252 \| $38 432 \| .37 \| $47 639 \| $37 793 \| .005 \| $62 138 \| $39 775 \| .02 \| \| Mean ratio (95% CI) \| 1.10 (0.89-1.35) \| Ref. \|  \| 1.26 (1.07-1.48) \| Ref. \|  \| 1.56 (1.07-2.28) \| Ref. \|  \| \| **ED cost** \|  \|  \|  \|  \|  \|  \|  \|  \|  \| \| **Individuals with ≥1 visit, No (%)** \| 266 (34.3) \| 771 (23.9) \|  \| 401 (44.6) \| 771 (23.9) \|  \| 89 (31.0) \| 771 (23.9) \|  \| \| Mean ($) \| $5335 \| $3737 \| <.001 \| $4442 \| $3764 \| .02 \| $5370 \| $3805 \| .008 \| \| Mean ratio (95% CI) \| 1.43 (1.19-1.71) \| Ref. \|  \| 1.18 (1.03-1.36) \| Ref. \|  \| 1.41 (1.09-1.82) \| Ref. \|  \| \| **Outpatient cost** \|  \|  \|  \|  \|  \|  \|  \|  \|  \| \| **Individuals with ≥1 visit, No. (%)** \| 775 (100.0) \| 3223 (100.0) \|  \| 900 (100.0) \| 3223 (100.0) \|  \| 287 (100.0) \| 3223 (100.0) \|  \| \| Mean ($) \| $12 816 \| $13 852 \| .12 \| $19 709 \| $13 117 \| <.001 \| $16 522 \| $13 479 \| .005 \| \| Mean ratio (95% CI) \| 0.93 (0.84-1.02) \| Ref. \|  \| 1.50 (1.38-1.64) \| Ref. \|  \| 1.23 (1.06-1.41) \| Ref. \|  \| \| **Pharmacy cost** \|  \|  \|  \|  \|  \|  \|  \|  \|  \| \| **Individuals with ≥1 prescription claim, No. (%)** \| 774 (99.9) \| 3028 (93.9) \|  \| 835 (92.8) \| 3028 (93.9) \|  \| 284 (99.0) \| 3028 (93.9) \|  \| \| Mean ($) \| $4573 \| $4835 \| .38 \| $4888 \| $4841 \| .87 \| $5534 \| $4887 \| .19 \| \| Mean ratio (95% CI) \| 0.95 (0.84-1.07) \| Ref. \|  \| 1.01 (0.90-1.13) \| Ref. \|  \| 1.13 (0.94-1.36) \| Ref. \|  \|   **Abbreviations:** ADRD, Alzheimer disease and related dementia disorders; ED, emergency department; MCI, mild cognitive impairment; Ref, reference  ^*^ All results were adjusted to December 2019 United States Dollars based on the Medical Care cost component of the Consumer Price Index and rounded to the nearest dollar.  ^†^ Means are estimated using a generalized linear model. |
| --- | --- | --- | --- | --- | --- | --- | --- | --- | --- | --- | --- | --- | --- | --- | --- | --- | --- | --- | --- | --- | --- | --- | --- | --- | --- | --- | --- | --- | --- | --- | --- | --- | --- | --- | --- | --- | --- | --- | --- | --- | --- | --- | --- | --- | --- | --- | --- | --- | --- | --- | --- | --- | --- | --- | --- | --- | --- | --- | --- | --- | --- | --- | --- | --- | --- | --- | --- | --- | --- | --- | --- | --- | --- | --- | --- | --- | --- | --- | --- | --- | --- | --- | --- | --- | --- | --- | --- | --- | --- | --- | --- | --- | --- | --- | --- | --- | --- | --- | --- | --- | --- | --- | --- | --- | --- | --- | --- | --- | --- | --- | --- | --- | --- | --- | --- | --- | --- | --- | --- | --- | --- | --- | --- | --- | --- | --- | --- | --- | --- | --- | --- | --- | --- | --- | --- | --- | --- | --- | --- | --- | --- | --- | --- | --- | --- | --- | --- | --- | --- | --- | --- | --- | --- | --- | --- | --- | --- | --- | --- | --- | --- | --- | --- | --- | --- | --- | --- | --- | --- | --- | --- | --- | --- | --- | --- | --- | --- | --- | --- | --- | --- | --- | --- | --- | --- | --- | --- | --- | --- | --- | --- | --- | --- | --- | --- | --- | --- | --- | --- | --- | --- | --- | --- | --- | --- | --- | --- | --- | --- | --- | --- | --- | --- | --- | --- | --- | --- | --- | --- | --- |
